# Activated platelets inhibit hepatocellular carcinoma cell differentiation and promote tumor progression via platelet-tumor cell binding

**DOI:** 10.18632/oncotarget.11300

**Published:** 2016-08-16

**Authors:** Rongfeng Zhang, Huishu Guo, Jingchao Xu, Bing Li, Yue-Jian Liu, Cheng Cheng, Chunyan Zhou, Yongfu Zhao, Yang Liu

**Affiliations:** ^1^ Institute of Heart and Vascular Diseases, First Affiliated Hospital of Dalian Medical University, Dalian, China; ^2^ Department of Central Laboratory, First Affiliated Hospital of Dalian Medical University, Dalian, China; ^3^ Department of General Surgery, Second Affiliated Hospital of Dalian Medical University, Dalian, China; ^4^ Department of Clinical Laboratory, First Affiliated Hospital of Dalian Medical University, Dalian, China; ^5^ Translational Research on Neurological Diseases Center, First Affiliated Hospital of Dalian Medical University, Dalian, China; ^6^ Department of Clinical Pharmacy, First Affiliated Hospital of Dalian Medical University, Dalian, China

**Keywords:** platelet, clopidogrel, tumor differentiation, hepatocellular carcinoma, TCF4

## Abstract

Lack of differentiation in hepatocellular carcinoma (HCC) is associated with increased circulating platelet size. We measured platelet activation and plasma adenosine diphosphate (ADP) levels in HCC patients based on differentiation status. Local platelet accumulation and platelet-hepatoma cell binding were measured using immunohistochemistry (IHC) or flow cytometry. Using a xenograft assay in NON/SCID mice, we tested the effects of the anti-platelet drug clopidogrel on platelet activation, platelet infiltration, platelet-tumor cell binding and tumor cell differentiation. HCC patients with poor differentiation status displayed elevated platelet activation and higher ADP levels. Platelets accumulated within poorly differentiated tissues and localized at hepatoma cell membranes. Platelet-tumor cell binding was existed in carcinoma tissues, largely mediated by P-selectin on platelets. NOD/SCID mice with xenograft tumors also exhibited increased platelet activation and platelet-tumor cell binding. Clopidogrel therapy triggered hepatoma cell differentiation by attenuating platelet activation and platelet-tumor cell binding. TCF4 knockdown promoted HepG-2 cell differentiation and inhibited tumor formation, and TCF4 could be the potential downstream target for clopidogrel therapy.

## INTRODUCTION

Hepatocellular carcinoma (HCC) is a major cause of cancer-related mortality worldwide [1]. Surgery is the primary intervention for early-stage HCC, but recurrence and metastasis rates are still very high in five years post-surgery [2]. Degree of differentiation is widely accepted as an important prognostic indicator of metastasis, recurrence and chemotherapy resistance in solid tumors, including HCC [3-6]. In clinical practice, more than 50% of HCCs are pathologically diagnosed as moderately or poorly differentiated carcinomas [7, 8].

Portal vein thrombosis (PVT) is a severe complication in HCC. Up to 40% of HCC patients have PVT at the time of diagnosis [9], and are more likely to experience metastasis and shortened survival compared to patients without PVT [10-12]. This indicates that platelets are hyper-activated in HCC. Increased platelet activation has been reported in HCC, pancreatic adenocarcinoma, gastric cancer and colon cancer, and is associated with increased tumor-node-metastasis (TNM) stage and risk of recurrence [13-16]. Activated platelets are reportedly involved in critical steps in cancer progression, including facilitating tumor cell growth and migration within vasculature [17].

Circulating platelets are heterogeneous in size, density and reactivity [18-20]. Large platelets are hyperactive in function, express high levels of membrane proteins, contain greater number of dense granules, release a large amount of adenosine diphosphate (ADP), and are more resistant to anti-platelet drugs [18, 19, 21]. Thus, mean platelet volume (MPV, a measurement of platelet size) and the proportion of large platelets are important hematological parameters that reflect platelet activation. In our preliminary retrospective study, we found that MPV and the proportion of large platelets in patients with poorly differentiated HCC were higher than in patients with well-differentiated tumors. We studied whether the anti-platelet drug, clopidogrel, could act as anti-cancer therapeutics by promoting tumor cell differentiation, independent of thrombus formation. We reported that tumor tissue-accumulated platelets induced platelet-tumor cell binding and inhibited tumor cell differentiation. Clopidogrel suppressed platelet-tumor cell binding and induced hepatoma cell differentiation in the xenograft model. These findings revealed the efficacy of anti-platelet drugs in HCC treatment.

## RESULTS

### Elevated platelet activation and circulating ADP levels in relation to HCC differentiation status

**Figure 1:**
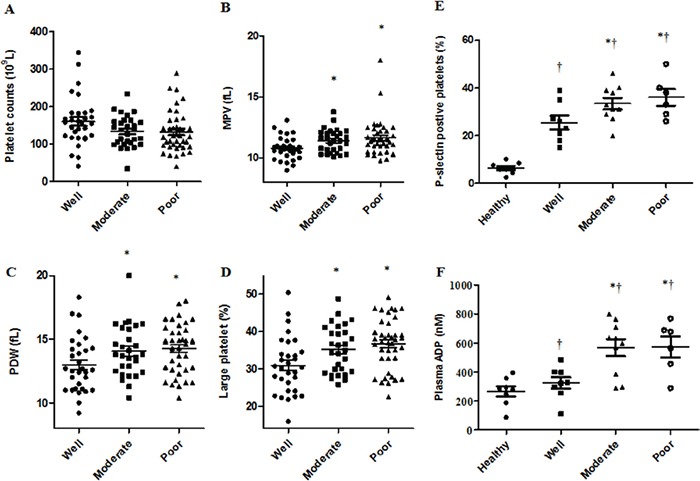
Elevated platelet activation and circulating ADP levels in relation to HCC patient cell differentiation status HCC patient platelet counts were the same among the three groups **A.** Increasing MPV **B.**, PDW **C.** and proportion of large platelets **D.** was observed in moderately and poorly differentiated compared with well differentiated disease. Patients with poor or moderate differentiation status had more P-selectin positive platelets and elevated ADP plasma levels compared to patients with well differentiated disease or healthy controls **E** & **F.** †*P*<0.05 vs. healthy controls, *P<0.05 vs. well differentiated.

Hematological parameters were determined in HCC tissue samples and patients were grouped by differentiation status. Patients with well and moderately differentiated tumors were older than those with poorly differentiated tumors (Table 1). Platelet count had no differences among the three groups, while MPV, platelet distribution width (PDW) and the proportion of large platelets were considerably greater in the moderately and poorly differentiated groups (Figure 1A–1D, Table 1). Patients with moderate and poor differentiation displayed high percentages of P-selectin positive platelets compared to those with well differentiated tumors or healthy controls (Figure 1E). Plasma levels of ADP in moderately/poorly differentiated patients increased by 2-fold compared with the other two groups (Figure 1F).

**Table 1:** HCC patient clinical and hematological parameters by differentiation status

	Well differentiated	Moderately differentiated	Poorly differentiated
Number	31	29	40
Male gender (%)	74	73	90
Age (year)	58 ±10	58±8	53±8^*^^†^
Platelet count (10^9^/L)	160.2±65.9	133.9±41.5	132.2±54.6
Mean platelet volume (fL)	10.8±0.9	11.4±0.9^*^	11.7±1.4^*^
Platelet distribution width (fL)	13.0±2.2	14.1±2.0^*^	14.3±1.9^*^
Large platelet (%)	30.9±7.6	35.2±6.3^*^	36.6±6.8^*^
White blood cell (10^9^/L)	6.0 ±2.6	5.7 ±2.4	5.6 ±1.74
Monocyte (10^9^/L)	0.5±0.2	0.4±0.2	0.5±0.2
Neutrophil (10^9^/L)	3.5±2.4	3.0±1.9	3.5±1.9
Lymphocyte (10^9^/L)	1.9±0.7	2.2±1.8^*^	1.5±0.5^*^^†^

Mean ± SD.

* *P*<0.05 vs. well differentiated,

† *P*<0.05 vs. moderately differentiated.

### Platelet accumulation within HCC tissues

**Figure 2:**
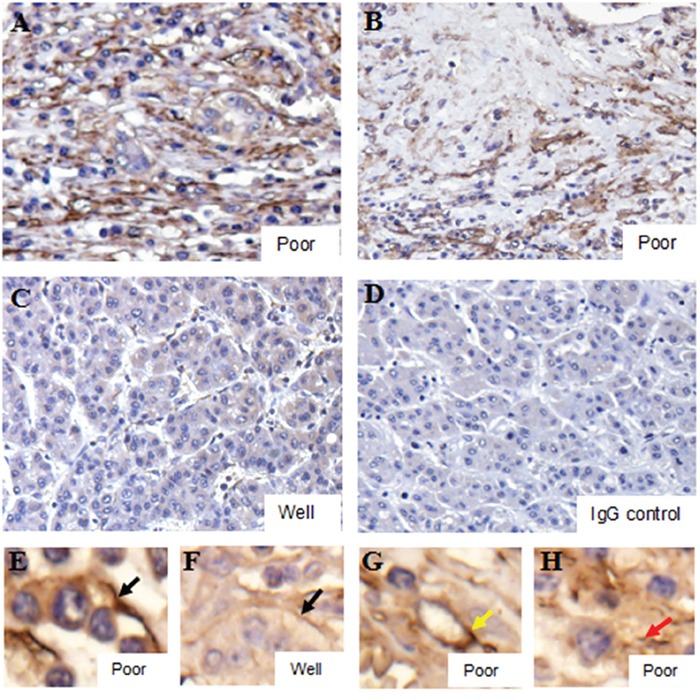
Platelet accumulation within HCC tissues is related to differentiation status Poorly differentiated tissues displayed prominent IHC platelet staining **A** & **B.**, with weak staining in well differentiated tissues **C.** Black arrows indicated co-localization of platelets and cell membranes **E** & **F.** Yellow and red arrows indicated platelets in intravascular regions **G.** and connective tissues **H.**, respectively. The IgG control was shown in **D.**

Immunohistochemistry (IHC) revealed platelets (CD41^+^) accumulated in HCC tissues (Figure 2A–2H). Poorly differentiated tissues displayed prominent platelet accumulation, while staining was reduced in well-differentiated tissues (Figure 2A–2C). In poorly differentiated tissues, platelets were largely found in the hepatoma cell membrane (Figure 2E), intravascular regions (Figure 2G) and connective tissues (Figure 2H). We thus hypothesized that platelets might bind to hepatoma cells.

### Platelet-tumor cell binding in tumor tissues and cell culture

**Figure 3:**
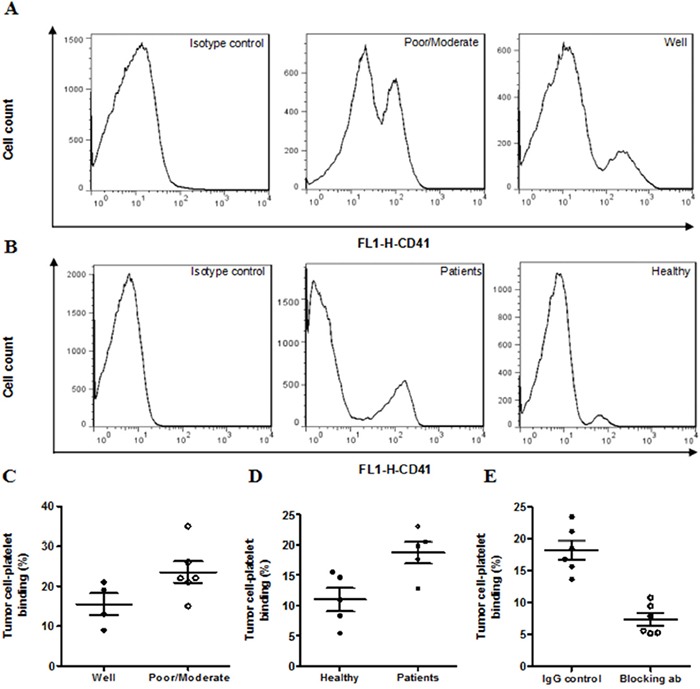
Platelet-tumor cell binding in HCC tissues and cell lines Representative FACS histogram showing platelet-tumor cell binding as determined by the population of cells expressing platelet marker CD41 in human HCC tissues **A.** or in HepG-2 cells co-cultured with human platelets **C.** Quantitative data were presented in panels **B** & **D.** *P<0.05, n=4–6/group.

We tested the degree of platelet binding to tumor cells in human HCC tissues and HepG-2 cell culture. Platelet-tumor cell binding increased by 40% in the poorly/moderately differentiated carcinoma tissues compared with well differentiated tissues (poorly/moderately: 23.2±3.4%; well: 15±2.8%, Figure 3A & 3C). We co-cultured HepG-2 cells with platelets isolated from healthy controls or HCC patients and found that platelets from HCC patients bind more frequently with HepG-2 cells than platelets from healthy controls (HCC patients: 18.7±1.7%; healthy control: 10.9±1.9%, Figure 3B & 3D). In the co-culture system, P-selectin blocking antibody abolished about 50% of platelet-tumor cell binding (Figure 3E), indicating that the binding was largely mediated by P-selectin on platelets.

### Anti-platelet intervention abolished tumor cell-evoked platelet activation and induced tumor cell differentiation in the mouse xenograft model

**Figure 4:**
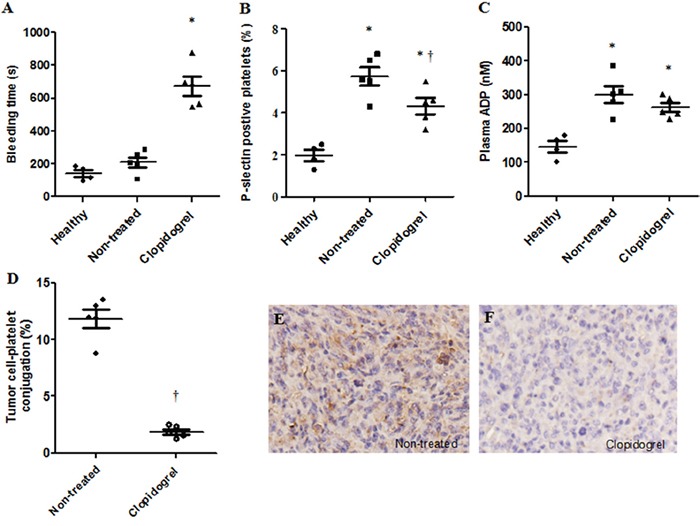
Anti-platelet intervention abolished tumor cell-evoked platelet activation, accumulation and binding in the mouse xenograft model Mice were injected with HepG-2 cells and one group was treated with clopidogrel. Tail bleeding time was measured in mice with or without treatment **A.** Increased P-selectin expression by circulating platelets, plasma ADP level and platelet-tumor cell binding were observed in xenograft mice and were inhibited by clopidogrel treatment **B–D.** Clopidogrel treatment prevented platelet accumulation in mouse tumor tissues **E** & **F.** *P<0.05 vs. healthy controls, †*P*<0.05 vs. non-treated, n=5/group.

**Figure 5:**
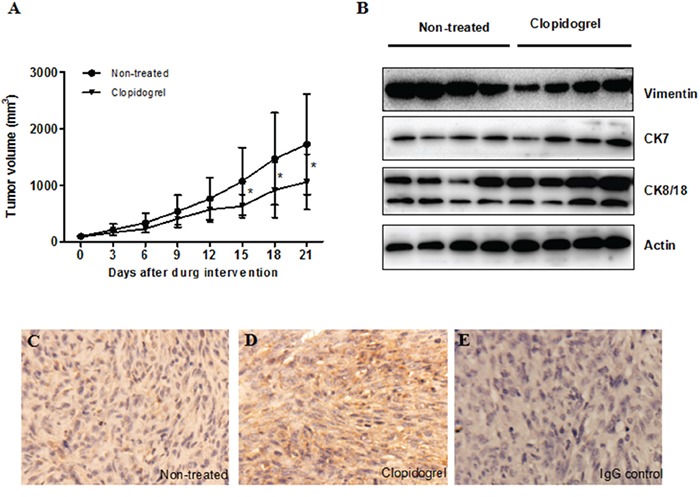
Anti-platelet intervention induced cell differentiation in the mouse xenograft model Tumor volume was measured every two days in both control and clopidogrel-treated mice **A.** Differentiation marker expression was analyzed by western blotting **B.** or IHC **C** & **D.** The IgG control was shown in **E.**

Similar to patients, mice with HepG-2 cell tumors had elevated P-selectin positive platelets and plasma ADP levels in comparison to healthy controls (Figure 4B & 4C). To determine whether platelets were involved in tumor progression, after three weeks of transplantation, mice received clopidogrel treatment for another three weeks. Clopidogrel efficacy was examined by measuring tail bleeding time. Prolonged tail bleeding was observed in the clopidogrel treatment group, indicating that the drug effectively inhibited platelets in our model (Figure 4A). In xenograft tumors, clopidogrel reduced the proportion of P-selectin positive platelets (non-treated: 5.7±0.4%; clopidogrel-treated: 4.3±0.5%, Figure 4B), the amount of platelet-tumor cell binding (non-treated: 11.8±0.8%; clopidogrel-treated: 1.9±0.2%, Figure 4D) and platelet local infiltration (Figure 4E & 4F). Clopidogrel treatment inhibited tumor growth from day 15 to day 21 compared with non-treated mice as indicated by tumor volumes (Figure 5A). Anti-platelet treatment induced CK8/CK18 and CK7 expression and reduced vimentin levels (Figure 5B), indicating that clopidogrel triggered hepatoma cell differentiation. This finding was confirmed by IHC showing that the abundance of heppar-1 (a specific HCC differentiation marker) in the non-treated group was decreased compared with the treatment group (Figure 5C–5E).

### Microarray analysis in xenograft tumors

**Figure 6:**
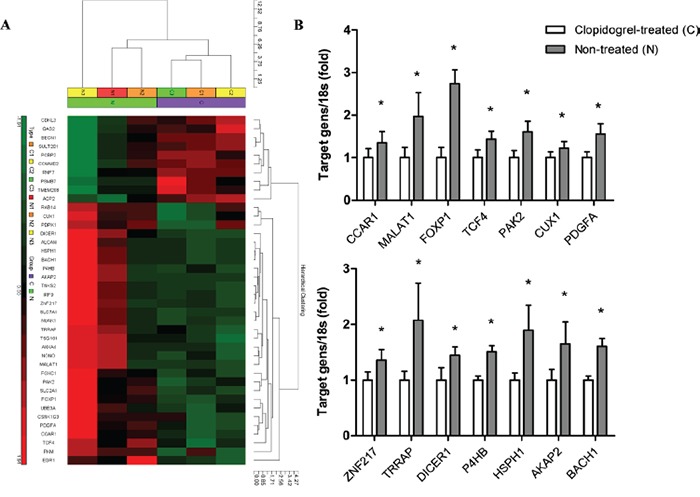
Microarray analysis in xenograft tumors from control and clopidogrel-treated mice An unbiased microarray expression profiling heat map between control and clopidogrel-treated mice was shown. High and low expression were indicated in red and green, respectively **A.** mRNA levels of 14 candidate genes in mouse tumor samples were verified with real-time PCR **B.** *P<0.05 vs. clopidogrel-treated.

Tumors were isolated from mice (n=3/group) and gene expression was measured via microarray analysis. A total of 214 genes were differentially expressed between the two groups. We found 140 genes were upregulated and 74 genes were downregulated in the non-treated group compared with the clopidogrel-treated group (Supplementary Table 1). Genes that have been extensively studied in tumor progression were identified and analyzed in a heat map (Figure 6A).

### Verification of gene expression by qPCR analysis

To validate the microarray data, 14 genes including *CCAR1, MALAT1, FOXP1, TCF4, PAK2, CUX1, PDGFA, ZNF217, TRRAP, DICER1, P4HB, HSPH1, AKAP2* and *BACH1* were analyzed via qPCR. Fold changes detected by qPCR were comparable with microarray results (Figure 6B).

### TCF4 knockdown induced differentiation and decreased tumorigenicity

**Figure 7:**
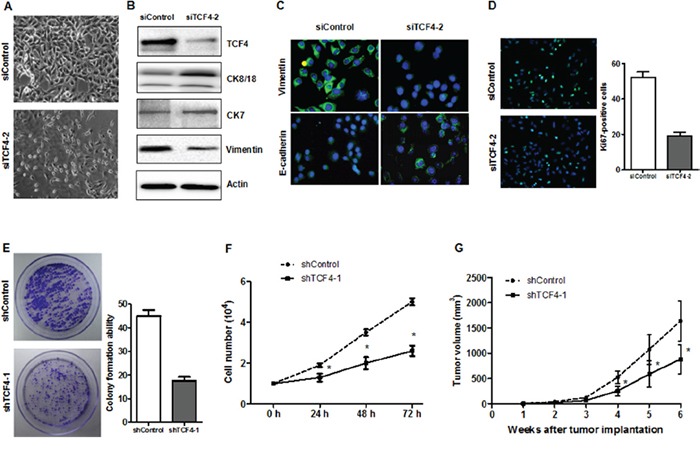
TCF4 knockdown induced cell differentiation and decreased tumorigenicity Phase contrast images of HepG-2 cells transfected with either siControl or siTC4 **A.** Differentiation marker expression was analyzed by western blotting **B.** or IF **C.** Cell proliferation was assessed by Ki-67 IF staining **D.**, proliferation assay **E.** and colony formation assay **F.** ShTCF4- or shControl-transfected cells were injected into NOD/SCID mice subcutaneously and tumor volumes were recorded **G.** *P<0.05 vs. control.

To explore whether TCF4 suppression inhibits HepG2 cell proliferation and leads to differentiation, cells were transfected with siTCF4/shTCF4 or control sequences. TCF4 knockdown in HepG2 cells led to marked morphologic changes compared with the control. In the TC4 knockdown group, CK8/18 and CK7 protein levels were increased, whereas vimentin level was reduced. Both cell number and colony formation assays showed that TCF4 knockdown inhibited cell growth (Figure 7D–7E), and suppressed the number of Ki67-postive cells (Figure 7F). *In vivo*, transfected HepG-2 cells exhibited reduced tumorigenicity compared with control cells (Figure 7G). These results suggested that TCF4 knockdown induced HepG-2 cell differentiation and reduced tumorigenicity.

## DISCUSSION

In the present study, we used both clinical data and xenograft assay in NOD/SCID mice to investigate the potential role of platelets in inhibiting hepatoma cell differentiation. Several novel findings have been made. *First*, an elevation in platelet activation and plasma levels of ADP was found in poor differentiation HCC in comparison to well differentiation. *Second*, platelets/platelet-tumor cell binding was observed in HCC tissues, with high levels in poorly differentiated tissues. *Third*, xenograft tumor induced an elevation in platelet activation and platelet-tumor cell binding in NOD/SCID mice. *Fourth*, clopidogrel therapy triggered hepatoma cell differentiation, with a marked attenuation of platelet activation and platelet-tumor cell binding as an important mechanism. *Fifth*, microarrays showed that a total of 214 genes were markedly changed in the tumor tissues after anti-platelet intervention, and we confirmed repression of TCF4 promoted hepatoma cell differentiation.

Platelets have been shown to mediate tumorigenicity via two fundamental mechanisms. First, platelet activation induces granular release of mediators, which directly support tumor growth and migration [22, 23]. Second, platelets can bind to different types of cancer cells, largely mediated by P-selectin on platelets and sialylated fucosylated carbohydrates on tumor cells [24-26]. Such binding protects tumor cells from immune attack or leads to tumor cell metastasis via epithelial-mesenchymal transition (EMT) [27, 28]. Platelets reportedly bind to breast cancer, melanoma, neuroblastoma, lung cancer, colon cancer and insulinoma cells [24, 27-31]. Consistent with this, we confirmed that HepG-2 cells bind platelets extracted from HCC patients, and less so to platelets from healthy persons, indicating that platelets from HCC patients were more activated. Further, increased platelet-tumor cell binding was detected in poorly differentiated as compared to well-differentiated HCC tissues, supporting our IHC results showing that platelets largely co-existed on hepatoma cell membranes. We speculated this binding would activate certain intracellular pathways and eventually prevent hepatoma cell differentiation. The hypothesis that platelet-tumor cell binding drives tumor cell escape into vasculature, promoting circulated tumor cells (CTC), requires further investigation.

It remains unclear how platelets are activated in cancer patients. P2Y receptors, including P2Y_1_ and P2Y_12_, are G-protein coupled receptors that control critical steps in platelet activation. ADP initially binds P2Y_1_ and induces transient activation of platelets, which is subsequently strengthened and sustained by interaction with P2Y_12_ [32, 33]. Generation of ADP has been documented in neuroblastoma, breast cancer and melanoma cells, and induces platelet activation [34, 35]. ADP depletion diminished tumor cell-induced platelet activation and was associated with reduced metastasis in mice [36]. In the present study, we demonstrated that plasma ADP levels were increased in HCC patients compared to healthy controls, and were higher in patients with well-differentiated compared to moderately or poorly differentiated disease. Similar results were observed in the mouse xenograft tumor model.

Inflammation also causes platelet activation. Most HCC cases are associated with hepatitis B-induced chronic inflammatory responses [37]. In a mouse model of chronic hepatitis B, Sitia, *et al.* showed that activated platelets contributed to acute hepatocellular injury-induced HCC by enhancing the accumulation of virus-specific CD8 T cells [38]. Clopidogrel combined with aspirin effectively prevented or delayed HCC progression and improved survival.

Tumor thrombosis is a severe cancer complication and forms the rationale for using anti-platelet drugs in cancer therapy. Besides inhibition of thrombus formation, clinical trials have documented broad benefits of anti-platelet drugs. As one of the most commonly used anti-platelet drugs, aspirin has been extensively studied in breast and colorectal cancer, demonstrating positive effects with respect to disease recurrence and patient mortality [39, 40]. Aspirin in combination with surgical treatment of non-small-cell lung cancer improved patient survival rates [41]. Moreover, aspirin decreased the proangiogenic effect of tamoxifen in breast cancer patients [42, 43]. In a retrospective analysis of prostate cancer, both aspirin and clopidogrel improved prognosis in patients undergoing radiation [44]. In a murine model, targeting of platelet membrane receptor GPIIb/IIIa, GPIb-IX-V or GPVI reduced pulmonary metastasis [45-49]. Clopidogrel reduced tumor growth and metastasis in a mouse model of pancreatic caner and breast cancer [50, 51]. In the present study, we demonstrated that clopidogrel treatment at a daily dosage of 5 mg/kg for three weeks induced tumor cell differentiation and inhibited tumor growth. The treatment in our study was started three weeks post-tumor implantation, and is thus applicable to clinical settings. In our study, we used only clopidogrel, and not aspirin, since clopidogrel was highly specific for platelets. Aspirin inhibits thromboxane A2 (TXA2) production by altering the activity of both cyclooxygenase-1 (COX-1) and COX-2, which are present in platelets, but also in other types of cells, including cancer cells.

A microarray assay was performed to identify potential anti-platelet intervention target candidates in hepatoma cells. A total 214 genes were differentially expressed between treated and non-treated cells, and about 40 genes involved in promoting cancer progression were upregulated in the non-treated group. Some, such as *CCAR1, MALAT1, FOXP1, TCF4* and *CUX1* were previously reported as highly expressed in HCC [52-56]. We demonstrated that TCF4 was a pivotal differentiation regulator in hepatoma cells, and TCF4 repression promoted HepG-2 cell differentiation and inhibited tumor formation. How platelet-tumor cell binding enhances TCF4 expression in hepatoma cells needs further investigation.

In conclusion, we found that platelets were highly activated in poorly differentiated HCC. Accumulation of activated platelets in HCC tissues induced platelet-tumor cell binding. Clopidogrel effectively inhibited platelet-tumor cell binding and promoted hepatoma cell differentiation. These results identify platelets as potential key regulators in HCC differentiation, supporting the use of anti-platelet therapy in cancer patients. Clopidogrel, as a common, inexpensive and safe intervention might constitute an effective adjuvant to chemotherapy or radiotherapy for both tumor inhibition and thrombosis prevention in HCC.

## MATERIALS AND METHODS

### Patient characteristics

We retrospectively analyzed 100 histologically diagnosed HCC patients who received a partial hepatectomy between January 2009 and December 2014 at the First Affiliated Hospital of Dalian Medical University. Patients with conditions that might affect platelet parameters, such as diabetes mellitus, cardiovascular diseases and anti-platelet drug use, were excluded from the study. Routine hematological assays examined prior to surgery were recorded. Fresh blood samples were obtained from 24 HCC patients prior to surgery and 8 healthy controls. Experimental protocols were approved by the ethics committee of the First Affiliated Hospital of Dalian Medical University.

### Determination of P-selectin positive platelets in human and mouse

Human venous blood samples were collected into a standardized tube containing EDTA as anticoagulant. Mouse blood was collected by cardiac puncture. To obtain platelet-rich plasma (PRP), blood (1 ml) was mixed with 300 μl of platelet washing buffer (pH 6.5, 4.3 mM K_2_HPO_4_, 4.3 mM Na_2_HPO_4_, 24.3 mM NaH_2_PO_4_, 113 mM NaCl, 5.5 mM glucose, 0.5% bovine serum albumin, 10 mM theophylline) and then centrifuged at 250 *g* for 2 min. Platelet pellets from PRP were collected by centrifugation at 2,000 *g* for 2 min and re-suspended in 1% fetal bovine serum (FBS) in PBS for flow cytometry. P-selectin antibody or isotype control (FITC-conjugated, BD Biosciences) was added to 10 μl of PRP and incubated for 30 min in darkness. P-selectin positive platelets were recorded and analyzed with a Becton-Dickinson FACSCalibur flow cytometer with FlowJo software.

### Assay for adenosine diphosphate (ADP) in plasma

ADP levels in patient and mouse plasma samples were determined using the ADP-Glo™ Kinase Assay kit (Promega) following the supplier's instructions.

### Cell culture

The human hepatoma cell line, HepG-2, was obtained from the Shanghai Cell Bank. Cells were maintained in DMEM (Gibco, high glucose) containing 10% FBS (Gibco) and cultured in a humidified 5% CO_2_ atmosphere at 37°C.

### Mouse xenograft assay and anti-platelet intervention

Six-week-old NOD/SCID mice were obtained from the animal center of Dalian Medical University and were maintained under specific pathogen free conditions. Mouse care and use protocols were approved by the Animal Committee of Dalian Medical University in accordance with national guidelines. HepG-2 cells (1×10^6^) were subcutaneously injected into the right flank. After transplantation, body weight and tumor size were measured every two days. For anti-platelet treatment, mice were randomly assigned into two groups and one group received clopidogrel treatment after three weeks transplantation. Tablets containing clopidogrel (75 mg) were ground into fine powder, freshly emulsified in 0.5% methyl cellulose solution and administered by gavage. A loading dose of 15 mg/kg was given at the first day of treatment, followed by the maintenance dose of 5 mg/kg once daily for the remainder of the three weeks [57]. The non-treated group was given 0.5% methyl cellulose solution by gavage.

### Bleeding time measurement

Inhibition of platelet activity by clopidogrel was tested by measuring tail bleeding time as previously reported [57]. Both treated and non-treated mice were subjected to bleeding time measurements. Sterile saline was poured into a 50 ml test tube and heated in a 37°C water bath for 1 h. A 10-mm segment of the tail tip was cut off and the tip of the tail was immersed in pre-warmed saline. Bleeding time was defined as the period of time where there was a clearly visible stream of blood that was continuously flowing.

### Western blotting

Mice were euthanized by an overdose of pentobarbital (100 mg/kg, intraperitoneal injection) after 6 weeks of transplantation. Tumors were removed and volumes were recorded. Western blotting was performed with primary antibodies for vimentin (1/500, Abcam), CK8/18 (1/1000, Cell signaling) and CK7 (1/2000, Cell signaling). Membranes were re-probed with β-actin to verity loading consistency.

### Immunofluorescence

Cells were seeded on poly-lysine pre-coated cover slides, fixed in 4% paraformaldehyde at room temperature for 20 min and then permeabilized in 1% Triton-X 100 for 10 min. After blocking in 5% of BSA, slides were incubated with primary antibody overnight at 4°C, followed by secondary antibody conjugated with Alex-488 for 30 min. Nuclei were stained with DAPI.

### Immunohistochemistry

IHC was used to determine the contents of platelets (CD41) or hepatocyte specific antigen (heppar-1) expression in tumor sections. Paraffin-embedded HCC tissue samples or mouse xenograft tumors were cut at a thickness of 5 μm and then mounted on coated microscope slides. Briefly, antigen retrieval was conducted by slide immersion in citrate-EDTA buffer followed by 5 min in a microwave oven at high power. Non-specific staining was blocked using 10% goat serum. After blocking, 50 μl of primary antibody (CD41, BD biosciences; heppar-1, Novus) was applied to each section for 1 h. A mouse IgG isotype control antibody (Jackson ImmunoResearch) was used at the same concentration as primary antibodies. After incubation with secondary antibody, sections were incubated with DAB until the desired staining developed. Sections were then counterstained with Myer's hematoxylin for 2 min, then dehydrated and mounted with DePex.

### Detection of platelet-tumor cell binding in tissues and cell culture

To assess conjugation between platelets and hepatoma cells, platelet marker CD41 positive tumor cells were measured in both cell culture and tumor samples using flow cytometry. Tissues from HCC patients and mice were mechanically dissociated using a scalpel, transferred to DMEM containing 1:1 collagenase I (3 mg/mL):hyaluronidase (100 U/mL; Sigma) and incubated at 37°C for 2 h on a shaking incubator. After filtration through a 40-μm filter, specimens were dissociated into single cells and washed with PBS several times. Cells were plated in DMEM with 10% FBS. After 2 h, fibroblasts were adhered. Un-adhered cells were collected by centrifugation and re-suspended in FACS buffer. Cells from human HCC tissues were incubated with anti-human CD41 (FITC-conjugated, BD Biosciences), anti-human CD45 (PE-conjugated, BD Biosciences) and anti-human CD31 (Alex Fluor 647-conjugated, BD Biosciences) for 30 min in darkness. Cells obtained from mice tumor specimens were incubated with anti-mouse CD41 (FITC-conjugated, BD Biosciences), anti-mouse CD45 (PE-conjugated, BD Biosciences) and anti-mouse CD31 (APC-conjugated, BD Biosciences). CD45 and CD31 antibodies were used to exclude leukocytes and endothelial cells in tissue samples.

Washed platelets from HCC patients and healthy controls were extracted as previously described. HepG-2 cells were co-cultured with platelets at 1:100 for 24 h [27]. P-selectin blocking antibody (25 μg/ml, R&D system) or IgG control antibody (BD Biosciences) was added to the co-culture. After 24 h, cells were harvested. re-suspended in FACS buffer and then incubated with anti-human CD41 antibody (FITC-conjugated, BD Biosciences). Cells were thoroughly washed and then analyzed using a FACSCalibur flow cytometer. Analysis was performed using FlowJo software. The percentage of CD41 positive cells in total hepatoma cells was determined.

### Microarray assay and comprehensive bioinformatics analysis

Total RNA was isolated with TRIzol (Invitrogen) according to the manufacturer's instructions. Gene expression profiling was performed using the Human Genome UI33 plus 2.0 array according to the manufacturer's instructions (Affymetrix). Fifteen micrograms of complementary RNA was fractionated and hybridized to an Affymetrix GeneChip.

### Validation of microarray data by qPCR analysis

Quantitative real-time PCR (qPCR) was used to verify the differential expression of 14 selected genes that were detected by microarray. These genes included *CCAR1, MALAT1, FOXP1, TCF4, PAK2, CUX1, TRRAP, PDGFA, ZNF217, DICER1, P4HB, HSPH1* and *AKAP2*. qPCR analysis was performed with a 7500 system, and primers are listed in Table 2. Cycling conditions included an initial, 5 min cycle at 95°C, followed by 40 cycles of 30 sec at 95°C, 30 sec at 54°C, and 15 sec at 72°C. Gene expression was quantified relative to 18S expression.

**Table 2:** Real-time PCR primers

Gene		Primer
18s	Forward	5′-TTGACGGGAAGGGCACCACCAG-3′
	Reverse	5′-GCACCACCACCCACGGAATCG-3′
CCAR1	Forward	5′-TCTCCCGAGGATACAAGCA-3′
	Reverse	5′-GACCAATGGGTAGGTGTAGAAA-3′
MALAT1	Forward	5′-CAGACCACCACAGGTTTACAGT-3′
	Reverse	5′-GACCATCCCAAAATGCTTCA-3′
FOXP1	Forward	5′-TCTCATAAACCATCAGCCCTCT-3′
	Reverse	5′-CCACTCATCTTCGTCTCAGCA-3′
TCF4	Forward	5′-ACAGAAAGGGGCTCATACTCA-3′
	Reverse	5′-CGAAAGGGTTCCTGGGTT-3′
PAK2	Forward	5′-CTTTTGGGAATGGAAGGATC-3′
	Reverse	5′-GCTTTCCGTGTAACCACCTCT-3′
CUX1	Forward	5′-CAGGCTGACTATGAAGAGGTGA-3′
	Reverse	5′-AGGTCGCTGTTGGAGATGC-3′
PDGFA	Forward	5′-GATACCTCGCCCATGTTCTG-3′
	Reverse	5′-TCAGGCTGGTGTCCAAAGA-3′
ZNF217	Forward	5′-CGCTGTTGTTCCATTCCG-3′
	Reverse	5′-CTGGTTCACAGAGGGTAGGC-3′
TRRAP	Forward	5′-CGACTTCCTCTACGACCACAT-3′
	Reverse	5′-CGACTCCTTCAGCATCTTCC-3′
DICER1	Forward	5′-CAGAACAGCATCCGCCACA-3′
	Reverse	5′-GCCTGTCCTTCTCCTCCTTGT-3′
P4HB	Forward	5′-TGCAAACAGTTGGCTCCC-3′
	Reverse	5′-TGCGTTCCCCGTTGTAATC-3′
HSPH1	Forward	5′-GAAAACAGCCTCAAGAAACCAG-3′
	Reverse	5′-CCGTAATTCAAAGCAACAGC-3′
AKAP2	Forward	5′-CACTGACTAATCCGAGACCACC-3′
	Reverse	5′-TTAGAAGGCTCGCTGTAGGG-3′
BACH1	Forward	5′-CACTGACTAATCCGAGACCACC-3′
	Reverse	5′-GAGTCGTCTCCCAAGCTAATG-3′

### Small interfering RNA transfection

RNA oligonucleotide double strands to target TCF4 were synthesized by GenePharma (siRNA-TCF4-1, 5′-CGAAAGUUUCCGAGACAAATT-3′; siRNA-TCF4-2, 5′-GAACCUAUCUCCAGAUGAATT-3′; siRNA-TCF4-3, 5′-GGAUUUAGCUGAUGUCAAATT-3′). Cells were seeded into six-well plates 24 h before transfection, with serum deprivation 1 h before transfection. In each well, 50 nM of siRNA and 5 μl of Lipofectamine 2000 (Invitrogen) were mixed in Opti-MEM (Gibco) and then added to cells. After 6 h of transfection, serum-free medium was replaced with medium containing 10% of FBS. RNAi efficiency was determined by western blotting at 48 h.

### Short-hairpin RNA transfection

shRNA constructs targeting TCF4 (shRNA-TCF4-1, CCGGGCAGACATCAATTCCAGTCTTCTCGAGAAGACTGGAATTGATGTCTGCTTTTT; shRNA-TCF4-2, CCGGCGAATTGAAGATCGTTTAGAACTCGAGTTCTAAACGATCTTCAATTCGTTT TT) were inserted into the pGPU6/GFP/Neo plasmid (GenePharma). HepG-2 cells were grown to 80% confluence prior to transfection. In each well, 4 μg of plasmid DNA and 10 μl of Lipofectamine 2000 (Invitrogen) were mixed in Opti-MEM (Gibco) and then added to cells. Medium was replaced 6 h post-transfection. Cells were selected with G418 (800 μg/ml). A non-target plasmid containing an shRNA that does not target human genes was used as a control.

### Cell proliferation assay

Cell proliferation was measured by cell number counting assay. We seeded 10,000 shControl- or shTCF4-transfected cells into a 24-well plate and counted for three continuous days to monitor cell growth.

### Colony formation assay

Approximately 1000 shControl- or shTCF4-transfected cells were seeded into 6-well plates in duplicate and incubated for 12 days. Cells were then fixed and stained with 0.4% crystal violet for 10 min at room temperature, and colonies were counted.

### Statistical analyses

Results are expressed as means ± SEM unless otherwise stated. ANOVAs followed by multiple comparison tests were performed using GraphPad Prism software. *P*<0.05 was considered statistically significant.

## SUPPLEMENTARY TABLE




