# Supplementary material for: Activated platelets inhibit hepatocellular carcinoma cell differentiation and promote tumor progression via platelet-tumor cell binding
Source: Oncotarget. 2016 Aug 16;7(37):60609–22. doi: 10.18632/oncotarget.11300 (PMC5312405; doi:10.18632/oncotarget.11300)
Supplement: Supplementary file 1 [file oncotarget-07-60609-s001.pdf]

Activated platelets inhibit hepatocellular carcinoma cell differentiation and promote tumor progression via platelet-tumor cell binding

SUPPLEMENTARY TABLE

Supplementary Table S1: Differentially expressed genes between non-treated group and clopidogrel-treated group examed by microarray.

This table listed each significantly deregulated/upregulated (fold change >1.5; p value < 0.05) gene. The EnsEMBL gene description, the associated gene name and information about the gene panel are available. N: non-treated group, C: clopidogrel-treated group.

See Supplementary File 1
